# Correlated multistate models for multiple processes: an application to renal disease progression in systemic *lupus erythematosus*

**DOI:** 10.1111/rssc.12257

**Published:** 2018-01-08

**Authors:** Aidan G. O’Keeffe, Li Su, Vernon T. Farewell

**Affiliations:** University College London, UK; University of Cambridge, UK

**Keywords:** Continuous time Markov model, Multistate model, Multivariate longitudinal data, Random effects

## Abstract

Bidirectional changes over time in the estimated glomerular filtration rate and in urine protein content are of interest for the treatment and management of patients with *lupus nephritis*. Although these processes may be modelled by separate multistate models, the processes are likely to be correlated within patients. Motivated by the *lupus nephritis* application, we develop a new multistate modelling framework where subject-specific random effects are introduced to account for the correlations both between the processes and within patients over time. Models are fitted by using bespoke code in standard statistical software. A variety of forms for the random effects are introduced and evaluated by using the data from the Systemic Lupus International Collaborating Clinics.

## 1 Introduction

Systemic *lupus erythematosus* (SLE) is a chronic autoimmune disease that affects multiple aspects of a person’s health, including skin condition, joint function and internal organs such as the kidney and neuropsychiatric systems. Because *lupus nephritis* (LN) is a cardinal feature of SLE, a recent study conducted by the Systemic Lupus International Collaborating Clinics (SLICC) aimed to investigate the bidirectional change over time in estimated glomerular filtration rate eGFR (the volume of blood that passes through the *glomeruli* of the kidney per minute) and proteinuria (urine protein content) in patients diagnosed with LN (Hanly *et al*., 2016). Since multistate models are well known as an approach to modelling processes with many discrete states that change over time (Hougaard, 1999; Andersen and Keiding, 2002; Meira-Machado *et al*., 2009), Hanly *et al*. (2016) separately modelled the eGFR and proteinuria processes in the SLICC data with two multistate models. The results of their analyses, such as the time spent in the different eGFR and proteinuria states, are useful in subsequent health economic analyses to inform decisions in managing LN for SLE patients in practice (Barber *et al*., 2018).

However, because both eGFR and proteinuria processes reflect patients’ renal function over time with different aspects for measurement, it is desirable to account for the within-subject correlation that is induced by the underlying kidney function when modelling these two processes. In this paper, motivated by the LN study on the SLICC data, we develop a correlated multistate model approach for multiple processes by incorporating subject level random effects (REs) in the modelling framework. The method that is developed allows the incorporation of REs in models where some or all states are recurrent.

RE models have been considered in survival data analysis methods, where they are commonly known as frailty models (Hougaard, 1984, 1995; Aalen, 1988). Usually, a subject level RE is introduced to act multiplicatively on the hazard functions in the survival models. For multistate models, REs have also been used to account for subject level heterogeneity (Aalen, 1987; Satten, 1999; Cook *et al*., 2004; Sutradhar and Cook, 2008; Yen *et al*., 2010; O’Keeffe *et al*., 2011, 2013; Joly *et al*., 2012). However, existing works have considered only specific and relatively simple *progressive* multistate models that do not contain cycles; in other words, they are for non-reversible processes where there is zero probability of returning to each non-absorbing state in the model. We are not aware of works on reversible multistate models with subject level random effects. This is partly because of the computational burden in fitting such complicated multistate models. In this paper, we aim to address this challenge and also consider more complex models for multiple processes.

Specifically, we develop a new class of correlated multistate models with subject level random effects for multiple reversible processes. Assuming a gamma distribution for the subject level random effects, the within-subject correlation over time for each of the multiple processes is taken into account in our models, which, to some extent, relaxes the Markov assumption that is taken in the ordinary reversible multistate models without random effects. Moreover, we allow for the within-subject correlation across multiple processes at fixed times, which is sensible in the LN study context because the underlying renal function induces such correlation for the observed processes of eGFR and proteinuria. On the basis of the scientific context of the LN study, we further explore different forms of the REs in modelling the eGFR and proteinuria processes and assess these models by comparing the empirical Bayes estimates of REs and other summary estimates (e.g. the time spent in different states in a fixed time period). The results of our analyses are useful to the subsequent economic modelling for the LN study.

The remainder of the paper is organized as follows. In Section 2 we describe the motivating SLICC data. The new class of multistate models with REs is introduced in Section 3. Section 4 describes the likelihood function and estimation procedure for fitting these models. The analysis results for the SLICC data are presented in Section 5 and we conclude with a discussion in Section 6.

## 2 The Systemic Lupus International Collaborating Clinics data

The SLICC comprise 32 academic medical centres across 11 countries and were established as an inception cohort for the long-term study of several outcomes in patients with SLE in October 1999 (Isenberg and Gladman, 2001). We focus on 568 patients from the SLICC inception cohort who have been diagnosed with LN and have at least two complete clinic visits before diagnosis of end stage renal disease or death up to December 2012. The clinic visits in the SLICC cohort are scheduled approximately annually. We calculated the mean time between consecutive visits for each of the 568 patients (a within-patient visit time summary measure) and the mean of these 568 within-patient values is 1.2 years with standard deviation 0.55 and interquartile range 1.00–1.24 years. As such, the time between visits does not vary considerably from patient to patient. In addition, the mean and standard deviation of the duration of follow-up are 5.2 and 3.1 years respectively. At each clinic visit several patient measurements are recorded, which include prescribed medications, lupus-related variables such as American College of Rheumatology classification criteria for SLE, the SLE disease activity index 2000, SLEDAI-2K, and the SLICC–American College of Rheumatology damage index, together with eGFR (in millilitres per minute per 1.73 m^2^) and proteinuria level PU (in grams per litre per day).

As in Hanly *et al*. (2016), we are interested in the change in the eGFR and PU levels over time for the SLICC patients. At any time point, each SLICC patient is assumed to stay in one of three states based on clinical categorizations of their eGFR and proteinuria level (Hanly *et al*., 2016). These states are numbered from 1 (the least severe category of eGFR or PU) to 3 (the most severe category of eGFR or PU). Table 1 shows the definitions of the eGFR and PU states and Table 2 presents some example data for eGFR and PU states recorded at clinic visits during the SLICC LN study.

Table 3 shows the observed transition matrices for the eGFR and PU states in the SLICC data. In general, there are fewer transitions between different states for eGFR than for PU at two consecutive clinic visits. The numbers of patients in each state at the start of observation are eGFR state 1, 504, eGFR state 2, 58, eGFR state 3, 6, and PU state 1, 244, PU state 2, 239, and PU state 3, 85, reflecting a range of disease severity at cohort entry across patients. We now consider the multistate models that will be used for the modelling of these eGFR and PU processes for the SLICC LN patient cohort.

## 3 Multistate models for eGFR and proteinuria

**Fig. 1:**
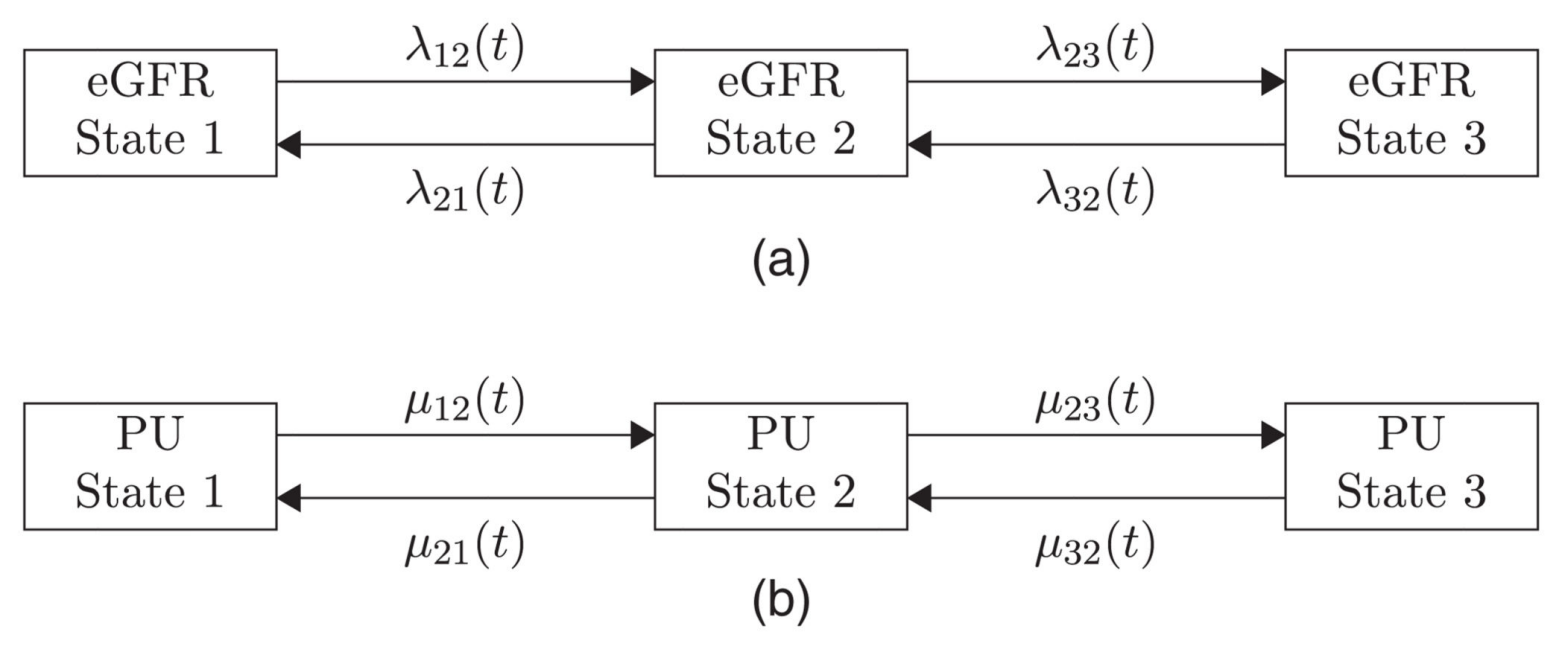
Diagram showing the paired multistate models for (a) the eGFR and (b) the PU processes: →, ←, possible transitions between states of the models

Movement by patients among the eGFR and PU states over time can be modelled by using multistate models (Hanly *et al*., 2016). Fig. 1 shows a pair of multistate models for eGFR and PU processes, with arrows showing permitted transitions between states. For each model, transitions between states are governed by a 3 × 3 matrix of ‘transition intensities’ and, for each model, the state space is {1, 2, 3} (since there are three states in each model). We define *λ_rs_*(*t*) and *μ_rs_*(*t*) to be the state *r* to state *s* transition intensities for the eGFR and PU models respectively ((*r*, *s*) ∊ {1, 2, 3} × {1, 2, 3}). Corresponding 3 × 3 matrices of transition intensities are denoted Λ(*t*) and *M*(*t*) where the (*r*, *s*) matrix entries are defined as *λ_rs_*(*t*) and *μ_rs_*(*t*) respectively.

In these multistate models, movements among eGFR and PU states are governed by underlying stochastic processes {*X*_eGFR_(*t*)} and {*X*_PU_(*t*), with corresponding filtrations ${ℱ}_{t-}^{\text{eGFR}}$ and ${ℱ}_{t-}^{\text{PU}}$ on some time interval T⊆[0,∞). Then, the state *r* to state *s* transition intensities are defined as

(1) $$\lambda_{rs}\left(t|{ℱ}_{t-}^{\text{eGFR}}\right)=\underset{\delta t↓0}{\text{lim}}\frac{1}{\delta t}\mathbb{P}\{X_{\text{eGFR}}(t+\delta t)=s|X_{\text{eGFR}}(t)=r,{ℱ}_{t-}^{\text{eGFR}}\},$$

(2) $$\mu_{rs}\left(t|{ℱ}_{t-}^{\text{PU}}\right)=\underset{\delta t↓0}{\text{lim}}\frac{1}{\delta t}\mathbb{P}\{X_{\text{PU}}(t+\delta t)=s|X_{\text{PU}}(t)=r,{ℱ}_{t-}^{\text{PU}}\}.$$

These transition intensities define the instantaneous rate of transition from eGFR or PU state *r* to state *s* and these may depend generally on states occupied by the system in the past through the dependence on ${ℱ}_{t-}^{\text{eGFR}}$ or ${ℱ}_{t-}^{\text{PU}}.$

### 3.1 The Markov assumption

As in Hanly *et al*. (2016), we make the common assumption that eGFR or PU states represent the states of two continuous time Markov chains. With this assumption, the future evolution of the eGFR process depends only on the current eGFR state (and likewise for the PU process) so that the dependences on past histories ${ℱ}_{t-}^{\text{eGFR}}$ and ${ℱ}_{t-}^{\text{PU}}$ may be removed from the transition intensities (1) and (2). This allows a likelihood function to be formulated easily for model fitting and facilitates calculations, such as times spent in states and predicted transition paths over time. To fit the multistate models for eGFR and PU, we consider the state *r* to state *s* transition probabilities, i.e., for some time *t*_2_ >*t*_1_, the probability that the eGFR or PU process is in state *s* at time *t*_2_, conditionally on that process having been in state *r* at time *t*_1_, denoted

$$\begin{array}{c} p_{rs}^{\text{eGFR}}(t_{1},t_{2})=\mathbb{P}\{X_{\text{eGFR}}(t_{2})=s|X_{\text{eGFR}}(t_{1})=r\};\\ p_{rs}^{\text{PU}}(t_{1},t_{2})=\mathbb{P}\{X_{\text{PU}}(t_{2})=s|X_{\text{PU}}(t_{1})=r\}. \end{array}$$

We then make an additional assumption that these Markov multistate models are time homogeneous, where a transition probability between times *t*_1_ and *t*_2_ depends on the length of the time interval *t*_2_ − *t*_1_ rather than the specific time values (*t*_1_, *t*_2_). As such, transition intensity matrices may be considered constant within a given time interval. For example, in an interval [0, *t*) we may define the eGFR and PU transition intensity matrices as Λ and *M*, with corresponding transition intensity matrices at time *t* given by exp (Λ*t*) and exp(*Mt*) respectively (Jackson, 2011). Here ‘exp’ denotes the matrix exponential for square matrices such that, for a square matrix *A*,

$$\text{exp}(A)=\sum_{k=0}^{\infty }\frac{A^{k}}{k!}.$$

R packages (R Development Core Team, 2008), e.g. mstate (Putter *et al*., 2006) and msm (Jackson, 2011), may be used to fit separate, uncorrelated, models for the eGFR and PU processes. As discussed in Section 1, in this paper we aim to introduce subject level random effects into this general multistate modelling framework, that includes reversible multistate models, to relax the Markov assumption, to reflect patient level heterogeneity better and to account for correlation between multiple processes.

### 3.2 Inclusion of random effects

We have defined two matrices that contain transition intensity parameters for each of the eGFR and PU processes, Λ(*t*) and *M*(*t*), where the (*r*, *s*) element of the corresponding matrix denotes the eGFR or PU state *r* to state *s* transition intensity at time *t*. Assuming that there are *N* subjects in the data (for the SLICC data *N* = 568), then for each subject we define an RE *U_i_* (for *i* ∊ {1, … , *N*}), where *U_i_* could be interpreted as an underlying propensity of the *i*th subject to move through the states of the models over time. Here, *U_i_* is a continuous random variable with support on (0, ∞).

In addition, we may define bijective functions of *U_i_* with the form $g_{rs}^{\left(j\right)}\left(U_{i}\right)$

$$g_{rs}^{\left(j\right)}:U_{i}\mapsto [0,\infty ),\;j\in \{1,2\}.$$

For simplicity of notation, *j* = 1 refers to the eGFR model and *j* = 2 refers to the PU model. Then, multiplying the (*r*, *s*) element of $\Lambda (t),\lambda_{rs}(t),\;\text{by}\;g_{rs}^{\left(1\right)}(U_{i}),$ we form a set of subject-specific transition intensities

$$\lambda_{rs}(U_{i},t)=g_{rs}^{\left(1\right)}(U_{i})\lambda_{rs}(t)$$

such that the subject-specific transition intensity matrix for the eGFR process is

$$\Lambda (t|U_{i})=\left(\begin{array}{ccc} -\lambda_{12}(U_{i},t) & \lambda_{12}(U_{i},t) & 0\\ \lambda_{21}(U_{i},t) & -\lambda_{21}(U_{i},t)-\lambda_{23}(U_{i},t) & \lambda_{23}(U_{i},t)\\ 0 & \lambda_{32}(U_{i},t) & -\lambda_{32}(U_{i},t) \end{array}\right).$$

Similarly, we can specify

$$\mu_{rs}(U_{i},t)=g_{rs}^{\left(2\right)}(U_{i})\mu_{rs}(t)$$

such that the subject-specific transition intensity matrix for the PU process is

$$M(t|U_{i})=\left(\begin{array}{ccc} -\mu_{12}(U_{i},t) & \mu_{12}(U_{i},t) & 0\\ \mu_{21}(U_{i},t) & -\mu_{21}(U_{i},t)-\mu_{23}(U_{i},t) & \mu_{23}(U_{i},t)\\ 0 & \mu_{32}(U_{i},t) & -\mu_{32}(U_{i},t) \end{array}\right).$$

$g_{rs}^{\left(j\right)}\left(U_{i}\right)$ can differ between the two processes and/or specific transitions, which enables a flexible approach to incorporating the subject-specific RE into the transition intensities. For example, it is probably sensible to assume that the SLICC patients who had higher deterioration transition intensities to move into more severe eGFR states (from state 1 to 2, and from state 2 to 3) were less likely to improve by moving from state 3 to state 2 or from state 2 to state 1. In other words, they tended to have lower improvement transition intensities. Therefore, we could specify $g_{rs}^{\left(1\right)}\left(U_{i}\right)$ to reflect such an inverse relationship between subject-specific deterioration and improvement transition intensities.

Incorporation of REs *U_i_* introduces both within-subject correlation over time for each individual process and the within-subject correlation across the two processes at fixed time points. This first correlation is useful to account for the remaining serial correlation after taking the Markov assumption for individual processes, whereas the second correlation, which is arguably more important, is reflecting the association of the eGFR and PU processes that is induced by the underlying renal function of the patients. In addition, as with other mixed effects models, the inclusion of REs accounts for unobserved heterogeneity between patients. This could be important when comparing this class of models with those without REs, especially if included explanatory variables have not truly reflected differences in the outcome processes between patients or differences in the number of observations made per patient.

In the next section, we outline the models that will be considered for the SLICC LN data by highlighting the different forms of $g_{rs}^{\left(1\right)}\left(U_{i}\right)$ and $g_{rs}^{\left(2\right)}\left(U_{i}\right)$ for REs. Throughout, for simplicity of notation, we assume that the fixed effects part of the transition intensity is time homogeneous, i.e.

$$\begin{array}{l} \lambda_{rs}(t)=\lambda_{rs},\\ \mu_{rs}(t)=\mu_{rs}. \end{array}$$

### 3.3 Different forms of random effects

#### 3.3.1 Model without random effects

First, a multistate model without REs can be used to model the eGFR and PU processes separately. Here we set $g_{rs}^{\left(1\right)}(U_{i})=g_{rs}^{\left(2\right)}(U_{i})=1$ and transition intensities for the eGFR and PU processes are given by *λ_rs_*(*U_i_*) = *λ_rs_* and *μ_rs_*(*U_i_*) = *μ_rs_* respectively. As discussed earlier, this model does not take into account the correlation between the eGFR and PU processes and the Markov assumption might not be sufficient to account for all within-subject serial correlations for each of the two processes.

#### 3.3.2 Simple random-effects model

Simple REs can be incorporated such that *U_i_* acts multiplicatively in the same manner on each baseline transition intensity by choosing $g_{rs}^{\left(1\right)}\left(U_{i}\right)=U_{i}\;\text{and}\;g_{rs}^{\left(2\right)}\left(U_{i}\right)=U_{i}.$ This simple RE model can be useful to characterize the phenomenon that the patients who had higher deterioration transition intensities would also have higher improvement intensities, i.e. patients were homogeneous in terms of how quickly they moved between states.

#### 3.3.3 Inverse random-effects model

As mentioned earlier, there could be an inverse relationship between subject-specific deterioration and improvement transition intensities. Therefore, in the inverse RE model, we assume that the RE *U_i_* acts differently on deterioration and improvement transition intensities. Specifically, *U_i_* acts multiplicatively on deterioration transitions, whereas the inverse 1/*U_i_* acts multiplicatively on improvement transitions, i.e. for *r* ∊ {1, 2}

$$\begin{array}{l} \lambda_{r,r+1}\left(U_{i}\right)=U_{i}\lambda_{r,r+1},\\ \lambda_{r+1,r}\left(U_{i}\right)=\frac{1}{U_{i}}\lambda_{r+1,r},\\ \mu_{r,r+1}\left(U_{i}\right)=U_{i}\mu_{r,r+1},\\ \mu_{r+1,r}\left(U_{i}\right)=\frac{1}{U_{i}}\mu_{r+1,r}, \end{array}$$

Here $g_{r,r+1}^{\left(1\right)}\left(U_{i}\right)=U_{i}\;\text{and}\;g_{r,r+1}^{\left(2\right)}\left(U_{i}\right)=U_{i},$ whereas $g_{r+1,r}^{\left(1\right)}\left(U_{i}\right)=1/U_{i}\;\text{and}\;g_{r+1,r}^{\left(2\right)}\left(U_{i}\right)=1/U_{i}.$

#### 3.3.4 Power inverse random-effects model

It is very possible that the RE acts on the eGFR and PU processes through the same functional forms but with different variabilities on the log-scale. Therefore, we relax the assumption in the inverse RE model and introduce the power inverse RE model, where a power transformation indexed by a new parameter *α* is applied to the RE when incorporated in the model for the PU process. Specifically, we choose $g_{r,r+1}^{\left(1\right)}(U_{i})=U_{i}\;\text{and}\;g_{r,r+1}^{\left(2\right)}(U_{i})=U_{i}^{\alpha },\;\text{and}\;g_{r+1,r}^{\left(1\right)}(U_{i})=1/U_{i}\;\text{and}\;g_{r+1,r}^{\left(2\right)}(U_{i})=1/U_{i}^{\alpha }\;\text{for}\;r\in \{1,2\}.$ Note that the parameter *α* ∊ ℝ needs to be estimated.

#### 3.3.5 Separate random-effects model

Finally, we could ignore the correlation between the eGFR and PU processes, and fit separate inverse RE models to the two processes. Specifically, for each patient we define two independent REs $U_{i}^{\left(1\right)}\;\text{and}\;U_{i}^{\left(2\right)}\left(i\in \left\{1,\ldots ,N\right\}\right).$ For *r* ∊ {1, 2}, we choose $g_{r,r+1}^{\left(1\right)}\left(U_{i}\right)=U_{i}^{\left(1\right)}\;\text{and}\;g_{r,r+1}^{\left(2\right)}\left(U_{i}\right)=U_{i}^{\left(2\right)},$ and $g_{r+1,r}^{\left(1\right)}\left(U_{i}\right)=1/U_{i}^{\left(1\right)}\;\text{and}\;g_{r+1,r}^{\left(2\right)}\left(U_{i}\right)=1/U_{i}^{\left(2\right)}.$ As discussed earlier, ignoring the correlation between the eGFR and PU processes that is introduced by the underlying renal function may not be desirable. In Section 5, we shall compare the results from different RE models fitted to the SLICC LN data and examine the model likelihoods as well as the corresponding empirical Bayes estimates of REs to evaluate the plausibility of different models based on the evidence from observed data.

## 4 Likelihood and estimation

### 4.1 Likelihood function

In the SLICC LN data, we have measurements over time on the eGFR and PU processes for each of 568 patients. We denote **t**_*i*_ = (*t_i_*_1_, … , *t_in_*_*i*_)^T^ to be the discrete time points at which the eGFR and PU states are recorded for the *i*th patient (with *n_i_ >* 1). Let ***λ*** and ***μ*** be the vectors of fixed baseline transition intensities for the eGFR and PU processes, where

$$\begin{array}{l} \lambda ={\left(\lambda_{12,}\lambda_{21,}\lambda_{23,}\lambda_{32}\right)}^{\text{T}},\\ \mu ={\left(\mu_{12,}\mu_{21,}\mu_{23,}\mu_{32}\right)}^{\text{T}}. \end{array}$$

In addition, we assume that the distribution of the RE of the *i*th patient is parameterized by ***θ*** with probability density function *f_U_i__*(*u_i_*, ***θ***). In this paper, we shall assume that *U_i_* has a Γ(1/*θ*, 1/*θ*) distribution for some *θ >* 0, which is a common choice for a frailty distribution in survival models (Clayton, 1978; Vaupel *et al*., 1979; Oakes, 1982; Henderson and Shimakura, 2003). Let *ϕ* = (***λ***, ***μ***)^T^ denote the collection of parameters that need to be estimated.

For each subject, we consider the states of each multistate model in continuous time as states of a Markov chain. The movement between states over time may be represented diagrammatically as a transition path of the form

$$X_{i}^{\left(j\right)}(t_{i1})\to \ldots \to X_{i}^{\left(j\right)}(t_{in_{i}})$$

where $X_{i}^{\left(j\right)}(t)$ is the random variable that is the state, for the *i*th patient in the *j*th multistate model (*j* = 1, eGFR model; *j* = 2, PU model) at time *t*. Under the Markov assumption, the probability of observing a particular transition path $\left(x_{i}^{\left(j\right)}(t_{i1}),\ldots ,x_{i}^{\left(j\right)}(t_{in_{i}})\right)$ in the *j*th multistate model, for the *i*th subject, is

$$\prod_{k=1}^{n_{i}-1}\mathbb{P}\left\{X_{i}^{\left(j\right)}(t_{ik+1})=x_{i}^{\left(j\right)}(t_{ik+1})|X_{i}^{\left(j\right)}(t_{ik})=x_{i}^{\left(j\right)}(t_{ik}),\phi ,u_{i}\right\}.$$

The joint likelihood for the *i*th patient for both the eGFR and the PU processes, given the RE *u_i_*, can be written as

(3) $$L_{i}(\phi |{\text{t}}_{i},u_{i})=\prod_{j=1}^{2}\prod_{k=1}^{n_{i}-1}\mathbb{P}\left\{X_{i}^{\left(j\right)}(t_{ik+1})=x_{i}^{\left(j\right)}(t_{ik+1})|X_{i}^{\left(j\right)}(t_{ik})=x_{i}^{\left(j\right)}(t_{ik}),\phi ,u_{i}\right\}.$$

The inclusion of *u_i_* allows a dependence between probabilities in the product (3). Integrating over the RE distribution, the overall contribution to the model likelihood function, from the *i*th patient, is

(4) $$L_{i}(\phi ,\theta |{\text{t}}_{i})=\int_{0}^{\infty }L_{i}(\phi |{\text{t}}_{i,}u_{i})f_{U_{i}}(u_{i},\theta )\text{d}u_{i}.$$

Finally, the likelihood function to be maximized for estimation is

(5) $$L(\phi ,\theta |\text{t})=\prod_{i=1}^{N}L_{i}(\phi ,\theta |{\text{t}}_{i})$$

where $\text{t}={\left({\text{t}}_{1}^{\text{T}},\ldots ,{\text{t}}_{N}^{\text{T}}\right)}^{\text{T}}.$

### 4.2 Estimation

The maximization of the likelihood function requires integration with respect to the RE *u_i_*. For some multistate models, usually where the state space is small and all states of the model are transient, it may be possible to perform this integration analytically. However, in general, it is necessary to use numerical integration to evaluate equation (4), especially in reversible multistate models.

To compute and maximize the likelihood function (5), we used the statistical software R (R Development Core Team, 2008). In particular, the msm package (Jackson, 2011) was used to compute the contributions from single subjects to the model likelihood function, given the REs *U_i_* (i.e. the expressions in equation (3)). Numerical integration in equation (4) was performed by using the integrate command. We considered a gamma-distributed RE and, when performing numerical integration, we transformed the RE by defining *v_i_* = exp (−*u_i_*) so that numerical integration could be performed over (0, 1] rather than [0, ∞), making the numerical integration step easier to implement.

Maximum likelihood estimates for model parameters, together with a numerically derived Hessian matrix, were obtained by using the Broyden–Fletcher–Goldfarb–Shanno optimization method (Broyden, 1970), implemented by using the optim command. The speed of the computation process is increased through the use of multicore programming via the parallel package. An outline of the R code that was used for the maximization of the likelihood function is provided in the on-line supporting information for this paper. In the next section, we fit previously described multistate models to the SLICC LN data and compare the inferences concerning the eGFR and PU processes over time in the SLICC LN patients.

## 5 Modelling renal disease progression in systemic *lupus erythematosus* patients

Using the models that were described in Section 3, we analysed the SLICC LN data and examined the bidirectional change over time in the eGFR and PU processes.

### 5.1 Model comparison

Table 4 summarizes estimated transition intensities as well as variance component estimates for REs from various fitted models.

It is not surprising that the transition intensities from the marginal model without REs are all smaller than those from the models with REs, because of the attenuation of marginal transition intensities in a similar manner to the difference between marginal and conditional covariate effects in the longitudinal data analysis literature (Diggle *et al*., 2002). There is a marked increase in the maximized log-likelihood between the marginal model and all RE models, which suggests that the introduction of REs into the multistate models leads to a better fit to the SLICC LN data.

Among the RE models, the inverse and power inverse RE models have the largest maximized likelihoods (−2367.93 and −2367.46 respectively) and smallest Akaike’s information criterion values. This suggests that the assumption of the inverse relationship between the deterioration and improvement transition intensities is better supported by the data. Moreover, the improvement of the fits of both inverse RE models compared with the separate RE model also indicates that there is evidence of underlying correlation between the eGFR and PU processes.

The power inverse RE model is an extended version of the inverse RE model, where the RE on the PU part of the model has the form $U_{i}^{\alpha }.$ In Table 4 we see that the estimate of *α* is 1.221 with corresponding 95% confidence interval (0.733, 1.708), which implies that *α* = 1 is a plausible value. This is also supported by the very similar maximized log-likelihoods from these two models. As a result, it is reasonable to assume that REs are not acting differently on the log-scale for the eGFR and PU processes. The transition intensity estimates and associated 95% confidence intervals are also very similar when comparing the inverse RE and power inverse RE models.

For the separate RE model, that assumes independence between the eGFR and PU processes, the PU transition intensity estimates (*μ*_12_, *μ*_21_, *μ*_23_, *μ*_32_) and RE variance estimate *θ*^(2)^ are very similar to the corresponding estimates in the inverse RE model. However, this is not so for the eGFR transition intensity estimates (*λ*_12_, *λ*_21_, *λ*_23_, *λ*_32_), which differ substantially from corresponding estimates in the inverse RE model. This can probably be explained by the different variance component estimates for the REs in the eGFR process. In the separate RE model, the estimate of *θ*^(1)^ is 1.213 whereas in the inverse RE model the estimate of *θ* is 0.549. Therefore, the eGFR transition intensity estimates and the corresponding confidence intervals in the separate RE model are inflated by the larger estimate of *θ*^(1)^. In contrast, the variance component estimate for the REs in the PU process, *θ*^(2)^, is 0.599, which is close to *θ* = 0.549 in the inverse RE model. Thus, the PU transition intensity estimates are similar between the two models. However, note that the variance component *θ*^(1)^ also has very wide 95% confidence interval (0.149, 9.85), probably because fewer transitions around the model space are observed for the eGFR process than for the PU process (see Table 3). In addition, a likelihood ratio test comparing this separate RE model with a separate RE model where the RE variances are constrained to be the same leads to a *χ*^2^ test statistic of 2.10 on 1 degree of freedom. Hence there is insuficient evidence to support different variances for the two RE distributions.

Overall, for the inverse RE model the maximized log-likelihood value is greater and the Akaike information criterion value is smaller when compared with the corresponding values for the separate RE models. As a result, although parameter estimates—particularly for PU transition parameters—do not differ substantially between these models, it is reasonable to assume that there is a level of dependence between the eGFR and PU processes and that a model with shared REs is preferable to either a model that includes separate REs or the marginal models (without REs) for eGFR and PU when modelling these data.

### 5.2 Empirical Bayes estimates of random effects

To compare different RE models further, we examined the empirical Bayes estimates of the REs from these models. The empirical Bayes estimates of the REs are given by

$$\begin{array}{l} {\hat{u}}_{i}=𝔼\left(U_{i}|\hat{\phi },\hat{\theta }\right)\\ \;=\frac{\int_{0}^{\infty }u_{i}f_{U_{i}}\left(u_{i},\hat{\theta }\right)L_{i}\left(\hat{\phi };\;u_{i},\;{\text{t}}_{i}\right)\text{d}u_{i}}{\int_{0}^{\infty }f_{U_{i}}\left(u_{i},\hat{\theta }\right)L_{i}\left(\hat{\phi };\;u_{i},\;{\text{t}}_{i}\right)\text{d}u_{i}}. \end{array}$$

**Fig. 2:**
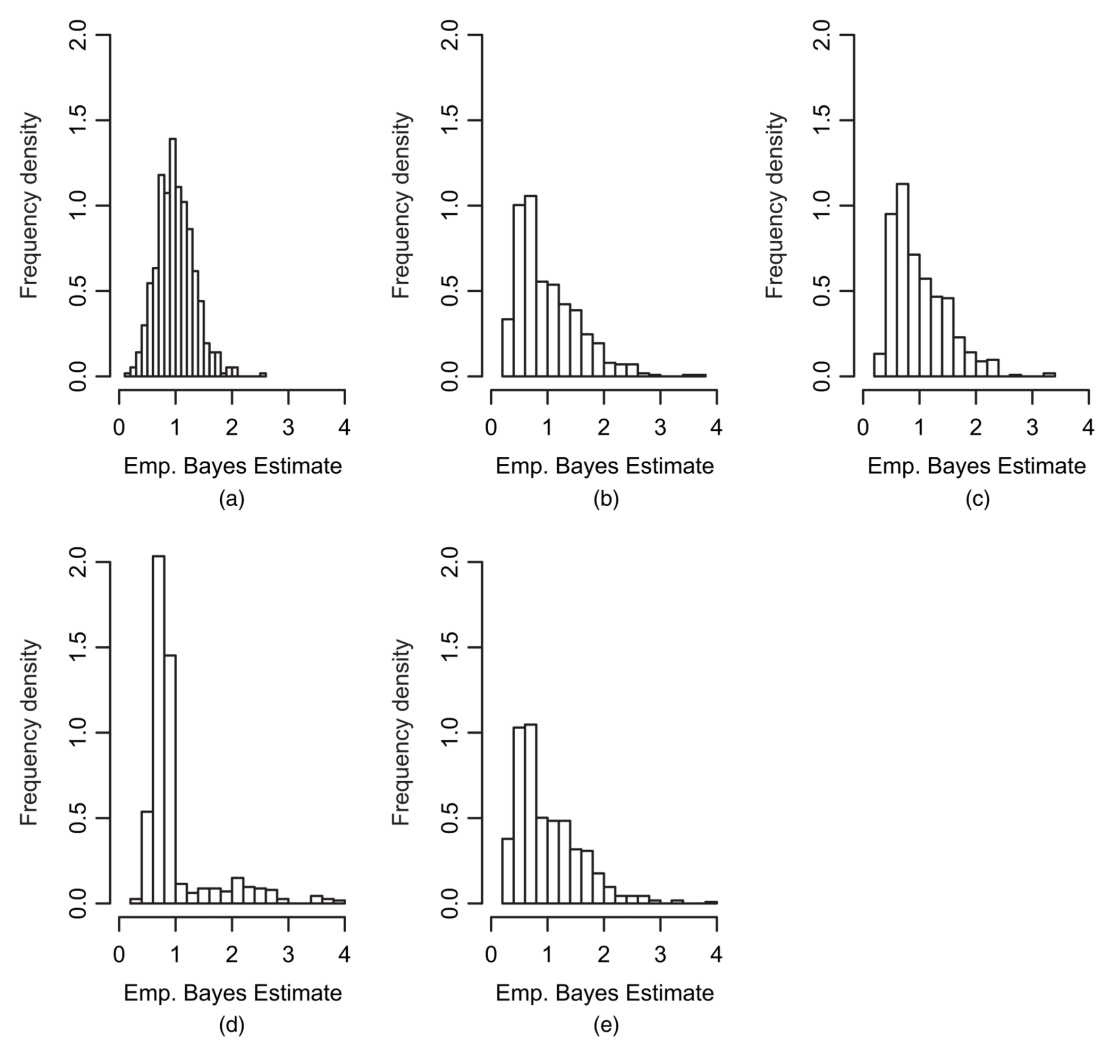
Histograms of the empirical Bayes estimates of the REs for the various RE models fitted to the SLICC LN data: (a) simple RE; (b) inverse RE; (c) power inverse RE; (d) separate RE, eGFR; (e) separate RE, PU

Here $L_{i}\left(\hat{\phi };u_{i},{\text{t}}_{i}\right)$ denotes the contribution to the model likelihood function from the *i*th patient in the SLICC LN data, evaluated at the corresponding model parameter estimates $\hat{\phi }.$ Fig. 2 shows histograms of the empirical Bayes estimates of the REs from each of the RE models (simple RE, inverse RE, power inverse RE and separate RE). Corresponding summary statistics are given in Table 5.

From Fig. 2 and Table 5, it is clear that the empirical Bayes estimates in the simple RE model, which assumes that REs act in the same manner on forward and backward transitions, are less variable than those in the inverse RE model. This is expected since the estimated RE variance (Table 4) is higher for the inverse RE model than for the simple RE model. The empirical Bayes estimates are very similar for the inverse RE and power inverse RE models. This is also not surprising, given the similarity of the fits of these two models in Table 4. Finally, when considering the separate RE model, Fig. 2 shows that the empirical Bayes estimates for the REs in the PU process are very similar to the overall empirical Bayes estimates in both the inverse and the power inverse RE models. In contrast, the histogram of empirical Bayes estimates for the REs in the eGFR process in the separate RE model (Fig. 2(d)) has a very different shape in which much of the mass occurs below 1 with much more right skewness. This suggests, consistent with estimated RE variances for these separate models, that variability between patients for the eGFR process is higher than for the PU process. However, as indicated earlier, the observed data also support a common level of variability because of the limited information that is available for the eGFR process.

Examining the data more closely, we find that, for the eGFR process, 451/568 patients (79.4%) are observed to stay in their initial eGFR state during the entire study follow-up. Conversely, for the PU process, 189/568 patients (33.3%) are observed to stay in their initial PU state at all clinic visits. This implies that patients tended to move frequently with respect to PU states rather than eGFR states, which was also noted in the recent analysis of the SLICC LN data using marginal multistate models by Hanly *et al*. (2016).

**Fig. 3:**
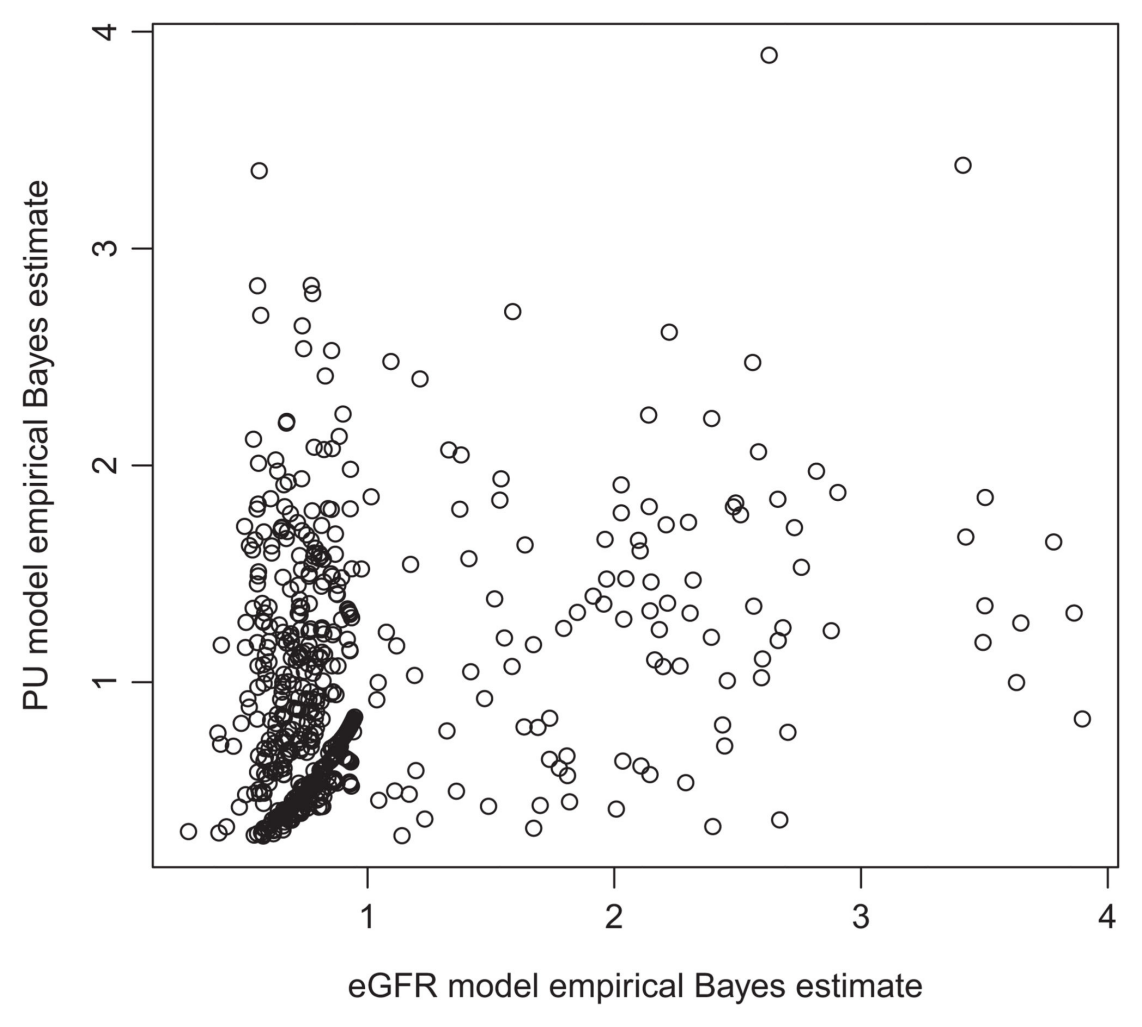
Scatter plot of the empirical Bayes estimates of the REs for the separate RE eGFR and PU models

As a further assessment of the level of within-subject correlation between the eGFR and PU processes, Fig. 3 shows a scatter plot of the empirical Bayes estimates of the REs for the separate RE eGFR and PU models. The Pearson estimate of linear correlation between these empirical Bayes estimates was calculated as 0.282. Although Fig. 3 does not show an obvious linear relationship between the estimated REs for all subjects, there is a clear cluster of points where smaller eGFR empirical Bayes RE estimates (typically values below 1) seem to coexist with smaller PU empirical Bayes RE estimates (also values below 1). We note that these models do not contain subject level explanatory variables and it is likely that, after the inclusion of extra variables, the variability in the empirical Bayes estimates would be reduced. Overall, it seems likely that there is a correlation between the eGFR and PU processes, in line with other results seen in this work and in Hanly *et al*. (2016).

### 5.3 Time spent in states in a fixed time period

Following Hanly *et al*. (2016), we also estimated the expected time spent in each state (for both eGFR and PU) over a fixed time period, which can feed into the subsequent economic modelling of the SLICC LN data.

Because the inverse RE model provides the best fit among all models that are under consideration, we shall use the fitted inverse RE model to generate the expected time spent in the eGFR and PU states in a 5-year period. For comparison, we also provide the results based on the marginal model, which was used in the analysis in Hanly *et al*. (2016).

On the basis of the fitted inverse RE model, we present two versions of the estimated expected time in states. One is by conditioning on REs *U_i_* = 1, which can be interpreted by the estimated expected time in eGFR and PU states for a typical patient in 5 years. Let $X_{i}^{\left(1\right)}(t)=r$ denote the current eGFR state for a given subject at time *t*. Conditionally on being in state *r*_1_, at time *t*_1_, the expected time spent in the state *r* over the period of time from *t*_1_ to *t*_1_ + 5 for a typical patient with *U_i_* = 1 is given by

$$\int_{t_{1}}^{t_{1}+5}\mathbb{P}\{X_{i}^{\left(1\right)}\left(t\right)=r|X_{i}^{\left(1\right)}\left(t_{1}\right)=r_{1},u_{i}=1\}\text{d}t.$$

The second version is obtained by calculating

$$\int_{t_{1}}^{t_{1}+5}\int_{0}^{\infty }\mathbb{P}\{X_{i}^{\left(1\right)}\left(t\right)=r|X_{i}^{\left(1\right)}\left(t_{1}\right)=r_{1},u_{i}\}f_{U_{i}}(u_{i};\hat{\theta })\;\text{d}u_{i}\;\text{d}t.$$

This double integration can be performed by using the adaptIntegrate command in the R package cubature (Narasimhan and Johnson, 2013). Alternatively, it can be done as follows:

(a) sample REs from the $\Gamma (1/\hat{\theta },1/\hat{\theta })$ distribution given the point estimate $\hat{\theta },$
(b) estimate the expected time in eGFR states given the sampled REs and
(c) calculate the sample averages of all expected times in the eGFR states across the RE samples.

The first version can be considered as the conditional estimates, whereas the second version is the marginal (population-averaged) estimates by averaging over the RE distribution. In this sense, the second version can be compared with the estimates from the marginal model without REs. Similar calculations can be done for the PU process as well.

Table 6 shows estimated times spent in the various states for eGFR and PU, conditional on initial states over a 5-year period, calculated by using these methods and beginning at some arbitrary time, owing to the assumption of time homogeneity (here, for specificity, *t*_1_ = 0). Examining Table 6, we see that, for patients who start at eGFR state 1, the marginal expected times in different eGFR states are similar for the inverse RE model and the marginal model. However, for patients who start at eGFR states 2 and 3, they have longer expected times in eGFR state 1 and shorter expected times in eGFR states 2 and 3 on the basis of the inverse RE model than for the marginal model (for initial state 2: 3.10 *versus* 2.66 years spent in state 1, 1.61 *versus* 1.94 years spent in state 2 and 0.29 *versus* 0.40 years spent in state 3; for initial state 3, 2.07 *versus* 1.19 years spent in state 1, 1.08 *versus* 1.23 years spent in state 2 and 1.85 *versus* 2.58 years spent in state 3). For the PU process, the expected times in the states are broadly similar between the two models, although we also note that the inverse RE model provides longer expected time in the PU state 1 when the initial state is 3 (2.52 *versus* 1.94 years).

Overall, the inverse RE model estimates suggest that patients were more likely to improve over time in terms of eGFR, compared with the estimates from the marginal model without REs. These results will lead to different cost estimates related to the eGFR states in the subsequent economic modelling, which suggests the significance in characterizing the heterogeneity between patients and accounting for correlation when modelling multiple processes of renal disease progression in SLE as developed in this paper. Indeed, for further health economic or cost-effectiveness analysis, including REs in multistate models to reflect heterogeneity could be advantageous in accounting for variation that is induced by this heterogeneity when compared with marginal models. This is in line with other approaches that incorporate uncertainty and patient level heterogeneity in such multistate models, e.g. model averaging (Jackson *et al*., 2009), probabilistic sensitivity analysis (Baio and Dawid, 2015) or Bayesian approaches (Baio, 2012).

Examination of the expected occupancy times for combinations of eGFR and PU states can also be done. Let $\left(X_{i}^{\left(1\right)}(t),X_{i}^{\left(2\right)}(t))=(r,s\right)$ denote the current *joint* eGFR and PU state for a given subject at time *t*. Conditionally on being in state (*r*_1_, *s*_1_) at time *t*_1_, the expected time spent in the state (*r*, *s*) over the period of time from *t*_1_ to *t*_1_ + 5 is given by

$$\int_{t_{1}}^{t_{1}+5}\text{ℙ}\{X_{i}^{\left(1\right)}\left(t\right)=r|X_{i}^{\left(1\right)}\left(t_{1}\right)=r_{1}\}\mathbb{P}\{X_{i}^{\left(2\right)}\left(t\right)=s|X_{i}^{\left(2\right)}\left(t_{1}\right)=s_{1}\}\text{d}t$$

from the marginal model and

$$\int_{t_{1}}^{t_{1}+5}\int_{0}^{\infty }\mathbb{P}\{X_{i}^{\left(1\right)}\left(t\right)=r|X_{i}^{\left(1\right)}\left(t_{1}\right)=r_{1},u_{i}\}\mathbb{P}\{X_{i}^{\left(2\right)}\left(t\right)=s|X_{i}^{\left(2\right)}\left(t_{1}\right)=s_{1},u_{i}\}\;f_{U_{i}}(u_{i}\text{;}\hat{\theta })\;\text{d}u_{i}\;\text{d}t$$

from the inverse RE model. This double integration was performed by using the adaptInteg rate command in the R package cubature (Narasimhan and Johnson, 2013).

Table 7 presents estimated expected times for the combined states based on the marginal model and the inverse RE model. In contrast with the expected times for each process separately (Table 6), estimating the time spent in the joint eGFR and PU state can lead to more detailed cost comparisons when the costs can be further categorized on the basis of the joint eGFR and PU state (Williams *et al*., 2017). This highlights the flexibility of our joint modelling approach as expected times spent in both joint and marginal (individual) states over a given period can be obtained to inform cost analyses. We also note that other common measures that are considered when fitting multistate Markov models, such as expected first-passage times, expected number of visits to a particular state within a given time period and mean sojourn times, can be easily computed within our modelling framework.

### 5.4 Further examination of the eGFR and PU processes

As a further comparison and examination of the relationship between the eGFR and PU processes, we fitted an inverse RE multistate model for the eGFR process, but with the PU state as an explanatory variable acting on eGFR transitions. Likewise, an inverse RE multistate model for the PU process was fitted with the eGFR state as an explanatory variable. The results from these fitted models are shown in Table 8.

In Table 8, for the eGFR model we see that deterioration transitions (eGFR state 1 → eGFR state 2 and eGFR state 2 → eGFR state 3) occur at a faster rate for subjects in higher PU states. Specifically, the estimated eGFR state 1 → eGFR state 2 transition intensities are, on average, exp(0.928) = 2.53 and exp(1.556) = 4.74 times higher for subjects in PU state 2 and PU state 3 respectively, when compared with those in PU state 1. Similarly, the estimated eGFR state 2 → eGFR state 3 transition intensities are, on average, exp(1.581) = 4.86 and exp(2.401) = 11.03 times higher for subjects in PU state 2 and PU state 3. The estimated log-intensity ratios do not suggest significant differences between subjects in different PU states on improvement eGFR state transitions.

In contrast, the log-intensity ratio estimates for the effect of eGFR state on the model for PU do not suggest that the eGFR state is significantly associated with PU state transitions. We note that the baseline PU state transition intensity estimates (*μ*_12_, *μ*_21_, *μ*_23_, *μ*_32_)^T^ in Table 8 are similar to those given in the separate RE model in Table 4. This may be expected since, for most subjects, the eGFR process is less variable over time when compared with the PU process. Therefore there are fewer changes in the eGFR state explanatory variable and less power to detect its relationship with the PU process. In addition, in these separate models dynamic covariates are assumed to be piecewise constant over time, which reflects a lagged relationship between the processes, whereas, in our joint model, the common RE reflects a cross-sectional correlation. Overall, consistent with the findings in Hanly *et al*. (2016), there is some evidence to suggest that the PU and eGFR processes are associated after accounting for other patients’ heterogeneities.

## 6 Discussion

In this paper, motivated by the application of modelling renal disease progression in patients with LN, we have developed methodology using subject-specific REs for correlated multistate models of multiple processes in continuous time. Data on related but different multiple processes are frequently collected in longitudinal studies but, within the multistate model framework, relatively little progress has been made on the use of REs to model such multiple processes, particularly for reversible processes. Motivated by the SLICC LN data, we developed multistate models with various forms of subject-specific REs for a pair of processes. This could be generalized to more than two processes and also to situations where the models are of different forms and do not contain the same numbers of states or transition patterns.

We have explored four different RE multistate models in the context of the SLICC LN data. When possible, the choice of forms of REs should be guided by the substantive knowledge regarding the disease processes of interest. In addition, the evidence based on the model likelihood and information criteria such as the Akaike information criterion and the Bayesian information criterion can be used for model selection.

A primary motivation for our work is to provide expected times in the eGFR and PU states in a fixed time period and to feed into subsequent economic modelling. Simple methods for calculating expected lengths of stay in various states are not applicable when dynamic covariates are included in the multistate modelling and, as such, much of the work in this paper was focused on models that did not explore associations between explanatory variables and renal disease progression, as was done in the clinical work of Hanly *et al*. (2016). However, in Section 5.4, we fitted two reversible multistate models that included dynamic explanatory variables to explore possible correlations between the eGFR and PU processes further. This also demonstrated that patient level and time varying explanatory variables can be easily incorporated in our modelling framework, as in standard multistate models without REs. We note that the computational time will be significantly longer when a large number of explanatory variables are included in the multistate models with REs. This is a common challenge which is shared by different non-linear models with REs in the literature. For multistate models particularly, care should be taken to ensure that the correlated models do not contain a large number of states, which introduces many parameters to be estimated. In addition, effects of explanatory variables could be constrained to be the same for different transitions if appropriate. We note that the numerical integration approach that we took for estimation may make it difficult to include more than one RE per patient, although different forms of a single RE are allowed as in this paper. In addition, the use of one RE for each patient facilitates the fitting of correlated multistate models to many processes by using our approach, where higher dimensional multivariate RE distributions may be difficult to consider computationally. This computational challenge is shared by other joint models for longitudinal data with REs (Rizopoulos, 2012).

The models that were considered in this paper can be implemented by using bespoke code run in standard statistical software and the code could easily be extended to other longitudinal or panel studies where multistate models accounting for subject heterogeneity are desirable given the context.

## Supplementary Material

Additional ‘supporting information’ may be found in the on-line version of this article:

Supplementary Material for “Correlated multi-state models for multiple processes: an application to renal disease progression in systemic lupus erythematosus”’.

Supplementary info

## Figures and Tables

**Table 1:** Clinical definitions of the eGFR and PU states

State	eGFR (ml min^−1^1.73 m^−2^)	PU (g l^−1^ day^−1^)
1	>60	<0.25
2	30–60	0.25–3.0
3	<30	>3.0

**Table 2:** Example SLICC data for the eGFR and PU states.

ptno	t (years)	eGFR state	PU state
001	0.00	1	2
001	1.14	1	2
001	2.17	2	2
001	3.05	2	3
⋮	⋮	⋮	⋮
002	0.00	1	1
002	1.54	1	1
002	2.97	1	2
⋮	⋮	⋮	⋮

**Table 3:** Numbers of observed transitions for eGFR and PU states between two consecutive clinic visits for the SLICC patients

From state	Numbers of transitions to the following states:		
	
	eGFR state 1	eGFR state 2	eGFR state 3
eGFR state 1	2303	95	5
eGFR state 2	86	136	21
eGFR state 3	1	10	26
	*PU state 1*	*PU state 2*	*PU state 3*
	
PU state 1	1167	257	20
PU state 2	355	547	56
PU state 3	45	85	59

**Table 4:** Estimated transition intensities (with corresponding 95% confidence intervals), variance component estimates for REs, maximized likelihood and Akaike information criterion values from fitted models for the SLICC LN data

	Parameter	Results for the following models:				
		
		Marginal	Simple RE	Inverse RE	Power inverse RE	Separate RE
eGFR parameters	*λ*_12_	0.051 (0.041, 0.062)	0.058 (0.046, 0.074)	0.053 (0.042, 0.066)	0.052 (0.042, 0.065)	0.068 (0.041, 0.112)
	*λ*_21_	0.461 (0.371, 0.573)	0.558 (0.421, 0.741)	0.496 (0.380, 0.649)	0.493 (0.382, 0.637)	0.682 (0.361, 1.288)
	*λ*_23_	0.112 (0.072, 0.174)	0.134 (0.082, 0.221)	0.073 (0.047, 0.116)	0.079 (0.049, 0.126)	0.052 (0.015, 0.181)
	*λ*_32_	0.346 (0.183, 0.652)	0.456 (0.212, 0.981)	0.453 (0.216, 0.952)	0.436 (0.212, 0.893)	0.775 (0.231, 2.600)
PU parameters	*μ*_12_	0.272 (0.239, 0.311)	0.332 (0.277, 0.398)	0.468 (0.385, 0.569)	0.464 (0.381, 0.567)	0.498 (0.401, 0.619)
	*μ*_21_	0.565 (0.504, 0.632)	0.679 (0.575, 0.801)	0.653 (0.551, 0.773)	0.687 (0.562, 0.839)	0.682 (0.572, 0.814)
	*μ*_23_	0.158 (0.120, 0.208)	0.239 (0.165, 0.346)	0.127 (0.091, 0.176)	0.119 (0.083, 0.170)	0.117 (0.084, 0.162)
	*μ*_32_	1.224 (0.988, 1.517)	1.916 (1.363, 2.693)	2.111 (1.550, 2.877)	2.291 (1.601, 3.278)	2.063 (1.513, 2.814)
RE variance	*θ*		0.462 (0.309, 0.690)	0.549 (0.417, 0.722)	0.415 (0.217, 0.793)	
	*θ*^(1)^					1.213 (0.149, 9.850)
	*θ*^(2)^					0.599 (0.433, 0.830)
Power RE model parameter	*α*				1.221 (0.733, 1.708)	
Maximized log-likelihood		−2448.96	−2418.42	−2367.93	−2367.46	−2382.88
Akaike information criterion value		4913.92	4854.84	4753.86	4754.92	4785.76

**Table 5:** Summary statistics of empirical Bayes estimates of the REs from the fitted RE models.

Model	Mean	Median	Standard deviation	Minimum	Maximum
Simple RE	1.00	0.96	0.33	0.19	2.58
Inverse RE	1.00	0.82	0.55	0.22	3.78
Power inverse RE	1.00	0.85	0.48	0.27	3.39
Separate RE: eGFR	1.00	0.79	0.62	0.27	3.90
Separate RE: PU	1.00	0.81	0.56	0.29	3.89

**Table 6:** Expected times spent in each of the eGFR and PU states, conditionally on the starting state over a 5-year period

Starting state	Expected time (years) spent in the following states:		
	
	eGFR state 1	eGFR state 2	eGFR state 3
*Marginal model (without REs)*			
eGFR state 1	4.67	0.29	0.04
eGFR state 2	2.66	1.94	0.40
eGFR state 3	1.19	1.23	2.58
	*PU state 1*	*PU state 2*	*PU state 3*
	
PU state 1	3.70	1.18	0.12
PU state 2	2.44	2.30	0.26
PU state 3	1.94	2.02	1.04
	*eGFR state 1*	*eGFR state 2*	*eGFR state 3*
	
*Conditional on U_i_* = *1*			
eGFR state 1	4.67	0.30	0.03
eGFR state 2	2.85	1.93	0.23
eGFR state 3	1.50	1.39	2.11
	*PU state 1*	*PU state 2*	*PU state 3*
	
PU state 1	3.24	1.67	0.09
PU state 2	2.33	2.53	0.14
PU state 3	2.06	2.34	0.60
	*eGFR state 1*	*eGFR state 2*	*eGFR state 3*
	
*Marginal—averaged over RE distribution*			
eGFR state 1	4.65	0.30	0.05
eGFR state 2	3.10	1.61	0.29
eGFR state 3	2.07	1.08	1.85
	*PU state 1*	*PU state 2*	*PU state 3*
	
PU state 1	3.44	1.39	0.17
PU state 2	2.72	2.05	0.23
PU state 3	2.52	1.83	0.65

**Table 7:** Expected times spent in each of the joint eGFR and PU states, conditionally on the starting state over a 5-year period.

Starting state	Expected time (years) spent in the following states (r,s) over a 5-year period:								
	
	(1, 1)	(1, 2)	(1, 3)	(2, 1)	(2, 2)	(2, 3)	(3, 1)	(3, 2)	(3, 3)
*Marginal model (without REs)*									
(1, 1)	3.47	1.09	0.11	0.21	0.08	0.01	0.03	0.01	0.00
(1, 2)	2.26	2.17	0.24	0.16	0.12	0.02	0.02	0.02	0.00
(1, 3)	1.78	1.88	1.00	0.14	0.12	0.04	0.02	0.02	0.00
(2, 1)	1.88	0.71	0.08	1.54	0.37	0.03	0.29	0.10	0.01
(2, 2)	1.46	1.06	0.14	0.77	1.07	0.10	0.21	0.17	0.02
(2, 3)	1.23	1.09	0.34	0.54	0.76	0.64	0.17	0.17	0.06
(3, 1)	0.82	0.34	0.04	0.88	0.31	0.03	2.00	0.53	0.05
(3, 2)	0.69	0.43	0.06	0.65	0.51	0.07	1.10	1.35	0.13
(3, 3)	0.61	0.47	0.11	0.53	0.52	0.19	0.80	1.03	0.75
*Inverse RE model (averaged over RE distribution)*									
(1, 1)	3.30	1.22	0.13	0.13	0.14	0.03	0.01	0.03	0.01
(1, 2)	2.60	1.87	0.19	0.11	0.16	0.03	0.01	0.03	0.01
(1, 3)	2.41	1.65	0.59	0.10	0.15	0.05	0.01	0.03	0.01
(2, 1)	2.30	0.74	0.07	1.03	0.51	0.07	0.11	0.14	0.03
(2, 2)	2.09	0.93	0.08	0.55	0.96	0.10	0.08	0.16	0.04
(2, 3)	2.01	0.94	0.15	0.43	0.74	0.43	0.07	0.15	0.06
(3, 1)	1.64	0.40	0.03	0.67	0.36	0.04	1.14	0.62	0.10
(3, 2)	1.57	0.46	0.03	0.54	0.49	0.06	0.61	1.10	0.14
(3, 3)	1.54	0.48	0.05	0.48	0.48	0.11	0.49	0.87	0.49

**Table 8:** Estimated transition intensities, RE variance and log-intensity explanatory variable effect estimates for eGFR and PU inverse RE multistate models with PU and eGFR states (respectively) acting as explanatory variables on model transition intensities.

Base transition intensity	Estimate (95% confidence interval)
*eGFR inverse RE model with PU states as explanatory variables*	
*λ*_12_	0.035 (0.024, 0.050)
*λ*_21_	0.666 (0.437, 1.014)
*λ*_23_	0.016 (0.008, 0.030)
*λ*_32_	0.383 (0.104, 1.419)

Log-intensity ratio	
PU state 2 on eGFR 1 → eGFR 2	0.928 (0.493, 1.364)
PU state 3 on eGFR 1 → eGFR 2	1.556 (0.890, 2.221)
PU state 2 on eGFR 2 → eGFR 1	−0.139 (−0.682, 0.405)
PU state 3 on eGFR 2 → eGFR 1	−0.141 (−1.012, 0.730)
PU state 2 on eGFR 2 → eGFR 3	1.581 (0.885, 2.277)
PU state 3 on eGFR 2 → eGFR 3	2.401 (1.354, 3.447)
PU state 2 on eGFR 3 → eGFR 2	0.913 (−0.554, 2.379)
PU state 3 on eGFR 3 → eGFR 2	0.266 (−1.897, 2.429)

RE variance *θ.*^(1)^	0.800 (0.710, 0.901)

*PU inverse RE model with eGFR states as explanatory variables*	
*μ*_12_	0.478 (0.383, 0.598)
*μ*_21_	0.688 (0.572, 0.827)
*μ*_23_	0.126 (0.089, 0.178)
*μ*_32_	2.121 (1.508, 2.983)

Log-intensity ratio	
eGFR state 2 on PU 1 → PU 2	0.255 (−0.396, 0.905)
eGFR state 3 on PU 1 → PU 2	1.856 (−2.308, 6.019)
eGFR state 2 on PU 2 → PU 1	−0.132 (−0.665, 0.402)
eGFR state 3 on PU 2 → PU 1	1.498 (−2.683, 5.680)
eGFR state 2 on PU 2 → PU 3	−1.293 (−2.550, −0.036)
eGFR state 3 on PU 2 → PU 3	1.655 (−2.003, 5.313)
eGFR state 2 on PU 3 → PU 2	−0.215 (−0.883, 0.453)
eGFR state 3 on PU 3 → PU 2	0.114 (−3.825, 4.053)
RE variance *θ*^(2)^	0.587 (0.428, 0.806)
