# Supplementary material for: Correlated multistate models for multiple processes: an application to renal disease progression in systemic lupus erythematosus
Source: J R Stat Soc Ser C Appl Stat. Author manuscript; Available in PMC 2019 May 15. (PMC6517283; doi:10.1111/rssc.12257)
Supplement: Supplementary info [file EMS82542-supplement-Supplementary_info.pdf]

Supporting Information for 'Correlated multi-state models for multiple processes: An application to renal disease progression in systemic lupus erythematosus'

Aidan G. O'Keeffe *[1], Li Su[2], and Vernon T. Farewell[2]

[1] *Department of Statistical Science, University College London, WC1E 6BT, UK*

[2] *MRC Biostatistics Unit, School of Clinical Medicine, University of Cambridge, Cambridge, CB2 0SR, UK*

1 R Code for the Maximisation of the Likelihood Function from Section 4

We present the R code that was used to evaluate the likelihood function for example presented in Section 5 of the manuscript. We assume that we have longitudinal data for a number of subjects across a number of ordered time points (`t`) such that the eGFR state (`gfstate`), proteinuria state (`pustate`) are recorded at each time point together with a unique patient identifier (`ptno`). Hence, the dataset is of the form given in Table 1

ptno	t	gfstate	pustate
001	0.00	1	2
001	1.14	1	2
001	2.17	2	2
001	3.05	2	3
⋮	⋮	⋮	⋮
002	0.00	1	1
002	1.54	1	1
002	2.97	1	2
⋮	⋮	⋮	⋮

Table 1: An example dataset using which the correlated models for renal function were fitted.

*E-mail address: `a.o'keeffe@ucl.ac.uk`; Corresponding author

The commented R code for the calculation of the likelihood function for the correlated eGFR and proteinuria models is given below. We note that this code will fit the 'simple random effects' model from Section 5 of the paper. Slight modifications can be made to the code to fit the inverse, power inverse and separate random effects models from the manuscript.

We make the following definitions for use in the presented R code:

- `v`= the log-transformed gamma random effect
- `data` = longitudinal dataset containing information on eGFR state and proteinuria level state, at the patient level, over time. Similar to that shown in Table 1.
- `Q_GF` = non-stochastic transition intensity matrix for eGFR.
- `Q_PU` = non-stochastic transition intensity matrix for proteinuria level.
- `theta` = random effect variance.

The function `'corr_gf_pu_msm1'`outputs patient-level likelihood function contribution as a function of the random effect, v, for user-supplied values of the random effect variance and transition intensity matrix parameters for eGFR and proteinuria level.

```
corr_gf_pu_msm1<-function(v,data,Q_GF,Q_PU,theta){

#Define tau<-1/theta

tau<-1/theta

#Form new transition intensity matrices Q1 and Q2 for eGFR
#and proteinuria models, repsectively. These new
#matrices will have random effects included.

Q1<-Q_GF
Q2<-Q_PU

#Multiply the relevant elements of Q1 and Q2 by the appropriate
#function of the random effect -log(v). Here -log(v) is a
#Gamma(tau,tau) random variable. v is in the range (0,1).

Q1[1,2]<--(log(v))*Q_GF[1,2]
Q1[2,3]<--(log(v))*Q_GF[2,3]
Q1[2,1]<--(log(v))*Q_GF[2,1]
Q1[3,2]<--(log(v))*Q_GF[3,2]
Q1[1,1]<--Q1[1,2]
Q1[2,2]<--Q1[2,1]-Q1[2,3]
Q1[3,3]<--Q1[3,2]

Q2[1,2]<--(log(v))*Q_PU[1,2]
```

```
Q2[2,3]<--(log(v))*Q_PU[2,3]
Q2[2,1]<--(log(v))*Q_PU[2,1]
Q2[3,2]<--(log(v))*Q_PU[3,2]
Q2[1,1]<--Q2[1,2]
Q2[2,2]<--Q2[2,1]-Q2[2,3]
Q2[3,3]<--Q2[3,2]

#Evaluate the patient-level contribution to the likelihood
#function from the eGFR model, for a
#given value of the random effect v. Here we use the msm
#library to evaluate the likelihood contribution. The
#outputted quantity from this step is-2*log-likelihood
#at the patient level, for a given value of v.

mod_GF<-msm(gfstate~t,data=data,opt.method="optim",method="BFGS",
control=list(maxit=0),qmatrix=Q1,hessian=FALSE,use.deriv=FALSE)$opt$value

#Evaluate the patient-level contribution to the likelihood
#function from the proteinuria level model, for a given value
#of the random effect v. Here we use the ‘msm’ library to
#evaluate the likelihood contribution. The outputted
#quantity from this step is -2*log-likelihood at the patient
#level, for a given value of v.

mod_PU<-msm(pustate~t,data=data,opt.method="optim",method="BFGS",
control=list(maxit=0),qmatrix=Q2,hessian=FALSE,use.deriv=FALSE)$opt$value

#We transform the -2*log-likelihood values from above to form
#likelihood function contributions from each of the eGFR
#and proteinuria level parts of the model, at the
#patient level and for a given value of the random effect, v.

lik_GF<-exp(-0.5*mod_GF)
lik_PU<-exp(-0.5*mod_PU)

#Compute the probability density function of v, for a given value
#of v and a given value of tau.

pdf<-(1/gamma(tau))*(1/v)*(tau^tau)*((-log(v))^(tau-1))*exp(-tau*(-log(v)))

#Multiply this evaluated probability density function by
#the patient-level likelihood function contributions from
#each of the eGFR and proteinuria level
#parts of the model.
```

```
out<-lik_GF*lik_PU*pdf

#Outputted below is a patient-level likelihood function
#contribution as a function of the random effect, v, for
#user-supplied values of the random effect variance and
#transition intensity matrix parameters  for eGFR
#and proteinuria level.

return(out)
}
```

The function below integrates takes the patient-level outputted likelihood function contribution from 'corr_gf_pu_msm1' and integrates out the random effect.

```
corr_gf_pu_msm3<-function(Q_GF,Q_PU,data,theta){

corr_gf_pu_msm2<-Vectorize(corr_gf_pu_msm1,vectorize.args=c("v"))
out<-integrate(corr_gf_pu_msm2,lower=0,upper=1,data=data,
theta=theta,Q_GF=Q_GF,Q_PU=Q_PU)$value
return(out)
}
```

The function below takes the following inputs:

- `pars` = a 9×1 vector of real-valued user-defined numbers. These are the numbers that will be transformed to transition intensity matrix entries and the random effect variance value during the likelihood maximisation process.
- `full_data` = a dataset containing longitudinal records of eGFR state and proteinuria state for all patients. Patients are indexed by the column 'ptno'.
- `ind`= patient number index.

```
corr_gf_pu_msm4<-function(pars,full_data,ind){

#Form the dataset 'dat' which is the subset of the full dataset,
#consisting of records from the patient for whom ptno = ind

dat<-subset(full_data,ptno==ind)

#Form the non-stochastic entries of the eGFR
#and proteinuria level transition intensity matrices using elements
#of the vector 'pars'

#(1,2) non-random entry of eGFR transition
#matrix:
```

```
Q12_GF<-exp(pars[2])

#(2,1) non-random entry of eGFR rate transition
#matrix:

Q21_GF<-exp(pars[3])

#(2,3) non-random entry of eGFR transition
#matrix:

Q23_GF<-exp(pars[4])

#(3,2) non-random entry of eGFR transition
#matrix:

Q32_GF<-exp(pars[5])

#(1,2) non-random entry of proteinuria level transition matrix:

Q12_PU<-exp(pars[6])

#(2,1) non-random entry of proteinuria level transition matrix:

Q21_PU<-exp(pars[7])

#(2,3) non-random entry of proteinuria level transition matrix:

Q23_PU<-exp(pars[8])

#(3,2) non-random entry of proteinuria level transition matrix:

Q32_PU<-exp(pars[9])

#Random Effect Variance
theta<-exp(pars[1])

#Form the non-stochastic eGFR and
#proteinuria level transition intensity matrices, for
#passing to 'corr_gf_pu_msm3'

#eGFR transition intensity matrix:

Q_GF<-rbind(c(-Q12_GF,Q12_GF,0),c(Q21_GF,-Q21_GF-Q23_GF,Q23_GF),c(0,Q32_GF,-Q32_GF))
```

```
#Proteinuria level transition intensity matrix:

Q_PU<-rbind(c(-Q12_PU,Q12_PU,0),c(Q21_PU,-Q21_PU-Q23_PU,Q23_PU),c(0,Q32_PU,-Q32_PU))

#Pass the patient-level data, eGFR
#transition intensity matrix, proteinuria level transition
#intensity matrix, random effect variance value and patient-level
#data to 'corr_gf_pu_msm3' for the formation of the patient-level
#contribution to the likelihood function and integration step.
#Return this patient-level likelihood function contribution.

lik_cont<-corr_gf_pu_msm3(Q_GF,Q_PU,dat,theta)
return(lik_cont)
}
```

The function `corr_gf_pu_msm5` evaluates each patient-level contribution to the likelihood function and outputs -2*log-likelihood function. The inputs are as follows

- `pts`= list of patient ids (ptno)
- `full_data`= a dataset containing longitudinal records of eGFR state and proteinuria state for all patients. Patients are indexed by the column `ptno`.
- `pars`= a 9x1 vector of real-valued user-defined numbers. These are the numbers that will be transformed to transition intensity matrix entries and the random effect variance value during the likelihood maximisation process.

```
corr_gf_pu_msm5<-function(pars,full_data,pts){

#Use mclapply command from the parallel library to calculate
#each patient-level contribution to the likelihood function


lik_conts<-mclapply(pts,corr_gf_pu_msm4,pars=pars,full_data=full_data,mc.cores=24)

#Take logs of patient-level likelihood contribution and sum
#to form the overall log-likelihood function

lik_conts<-as.vector(unlist(lik_conts))
log_lik<-log(lik_conts)

#Return -2*(overall log-likelihood function)

out<--2*sum(log_lik)
return(out)
```

```
}
```

The function 'corr_gf_pu_msm5' is passed to the optim function within R so that $-2 \times$ log-likelihood is minimised (thereby maximising the likelihood function) for given parameter starting values 'pars'.
